# Articles of Food That Are Laxative

**Published:** 1887-06

**Authors:** 


ARTICLES OF FOOD THAT ARE LAXATIVE.—Cracked wheat, oatmeal and Indian meal gruel, Graham bread, bran bread (¾ flour, ¼ bran), new potatoes, green corn, turnips, onions, apple sauce, rhubarb and gooseberries, stewed prunes, honey, molasses candy, fresh fruits (oranges, apples, pears, peaches, plums, apricots), dried fruits (raisins, figs, prunes, dates, tamarinds, dried apples, etc.), salad oil, porter, ale, cider, mineral water.—*Technics.*

